# F229. THE BIOLOGICAL UNDERPINNINGS OF TREATMENT RESPONSE IN DELUSIONAL DISORDER: A SYSTEMATIC REVIEW OF QUALITATIVE EVIDENCE-TO-DATE

**DOI:** 10.1093/schbul/sby017.760

**Published:** 2018-04-01

**Authors:** Alexandre González-Rodríguez, Francesc Estrada, Itziar Montalvo, José Antonio Monreal, Diego Palao, Javier Labad

**Affiliations:** 1 Parc Tauli University Hospital, Institut d’Investigació i Innovació Parc Taulí (I3PT); 2 Parc Tauli University Hospital; 3 Parc Tauli University Hospital, Institut d’Investigació i Innovació Parc Taulí (I3PT), Autonomous University of Barcelona (UAB)

## Abstract

**Background:**

The dopamine hypothesis of schizophrenia has been extensively proposed as a neurobiological mechanism that explains the relationship between schizophrenic symptoms and hyperdopaminergic states. This hypothesis is supported by direct and indirect evidence, and it mainly postulates that antipsychotics act blocking dopamine receptors. When focusing on delusional disorder patients, especially delusional disorder somatic type, a great effort towards the search for a biological basis of treatment response has been recently demonstrated. Thus, the main goal of this systematic review was to examine the evidence explaining the biological underpinnings of treatment response in delusional disorder.

**Methods:**

A systematic review was performed using Pubmed, Scopus and PsycINFO databases (from 1990 to October 2017), according to the Preferred Reporting Items for Systematic Reviews and Meta-Analyses (PRISMA) statement. The following search terms were used: [(‘treat*’ OR ‘therap*’ OR ‘biol*’) AND (‘delusional disorder’)]. This systematic computerized search was completed by additional studies hand-checked through reference lists from the included studies and review articles. Studies were only included if the met our inclusion criteria: (a) the International Classification of Diseases (ICD) or Diagnostic and Statistical Manual of Mental Disorders (DSM) diagnosis for delusional disorder, (b) be published in peer-reviewed journals, (c) in English, German or Spanish, (d) and reporting a hypothesis for the biological basis of treatment response in delusional disorder, irrespective of method and study design. Exclusion criteria were: (a) studies including organic delusional disorder or (b) somatic delusions secondary to other psychiatric diagnoses. The literature search strategy, data extraction and synthesis was conducted independently by two authors (A.G.R, F.E.). When disagreement, it was solved by consensus.

**Results:**

A total of 59 articles were identified, of which 12 met our inclusion criteria. Four hypotheses were addressed: (1) Dopaminergic dysfunction (n=4): ziprasidone-induced supersensitivity psychosis by chronic blockade of D2 Dopamine Receptor (DRD2) (n=1); pretreatment levels of plasma homovallinic acid (pHVA) (n=1); dopamine transporter (DAT) dysfunction (n=1) and effectiveness of aripiprazole (DRD2 agonist) (n=1). (2) Serotonergic dysfunction (n=6): drug occupancy in 5-HT1A and 5-HT2A receptors (n=3) and efficacy of 5-HT2 antagonists (n=3). Brain dysfunction (n=7): hypoperfusion in cerebral blood flow in temporal and parietal lobes, left side (n=5), right side (n=1) and lack of basal ganglia and subcortical gray matter lesions (n=1). Genetic evidence (n=1): implications of DRD2 Ser311Cys, DRD3 Ser9Gly and TH VNTR polymorphisms.

**Discussion:**

The strongests biological contributors for treatment response in delusional disorder seem to be those implicating monoaminergic systems, particularly dopamine and serotonergic neurotransmitters. Although the low level of evidence, the serotonergic dysfunction may be associated with response rates, especially in delusional disorder somatic type. The link between genetic variants of dopamine receptors and neuroimaging findings in delusional disorder may open new avenues for the search of the biological underpinnings of treatment response. The evidence for an integrated model involving dopamine and serotonin systems bears further investigations.

